# A rare case of multiple endocrine neoplasia type 1 initially presenting as an asymptomatic, huge mediastinal mass: case report

**DOI:** 10.1186/s12902-021-00695-9

**Published:** 2021-02-25

**Authors:** Ji Eun Jun, You-Cheol Hwang, Kyu Jeong Ahn, Ho Yeon Chung, In-Kyung Jeong

**Affiliations:** Department of Endocrinology and Metabolism, Kyung Hee University Hospital at Gangdong, Kyung Hee University School of Medicine, 892 Dongnam-ro, Gangdong-gu, Seoul, 05278 Republic of Korea

**Keywords:** Case report, Multiple endocrine neoplasia, MEN1, Thymic carcinoid tumor

## Abstract

**Background:**

Multiple endocrine neoplasia type 1 (MEN1) is a rare inherited syndrome that concurrently involves various endocrine glands. We report a rare case of MEN1 in a 43-year-old man whose first manifestation was an asymptomatic mediastinal mass.

**Case presentation:**

A 13-cm-sized mediastinal mass was diagnosed as an atypical thymic carcinoid by computed tomography and percutaneous needle biopsy. In addition, hypercalcemia from a left inferior parathyroid hyperplasia, and a non-functioning gastric neuroendocrine tumor seen on esophagogastroduodenoscopy were found. Therefore, the patient was clinically diagnosed with MEN1 syndrome, and underwent surgical resection of thymic carcinoid tumor after pre-operative concurrent chemoradiation therapy to decrease tumor size and volume. Parathyroid lesion and gastric neuroendocrine tumor were also removed. Finally, a *MEN1* gene mutation was observed in the patient and his 7-year-old son.

**Conclusion:**

Despite its rare occurrence, thymic carcinoid tumor should be considered as a MEN1-associated tumor and necessitates screening of other endocrine glands. Thymic carcinoid tumor carries a poor prognosis in patients with MEN1, and thus it needs to be carefully monitored even after radical excision.

**Supplementary Information:**

The online version contains supplementary material available at 10.1186/s12902-021-00695-9.

## Background

Multiple endocrine neoplasia type 1 (MEN 1) is a rare hereditary syndrome characterized by a predisposition for neoplasms involving two or more endocrine glands, primarily of parathyroid, anterior pituitary, and enteropancreatic origin [1]. Other endocrine tumors of MEN1 include carcinoid tumors, adrenocortical tumors, and rarely, pheochromocytoma [2]. Among them, thymic carcinoid tumor is less common, with an incidence of 3.6–8.4%, and only 25% of all thymic carcinoid tumors occur in patients with MEN1 [3]. Despite its low incidence, MEN1-associated thymic carcinoid tumor leads to poor prognosis due to its more aggressive nature and strong potential for metastasis [4, 5]. Herein, we report a case of MEN1 in a patient with an asymptomatic and huge mediastinal mass as an initial presentation.

## Case presentation

**Fig. 1:**
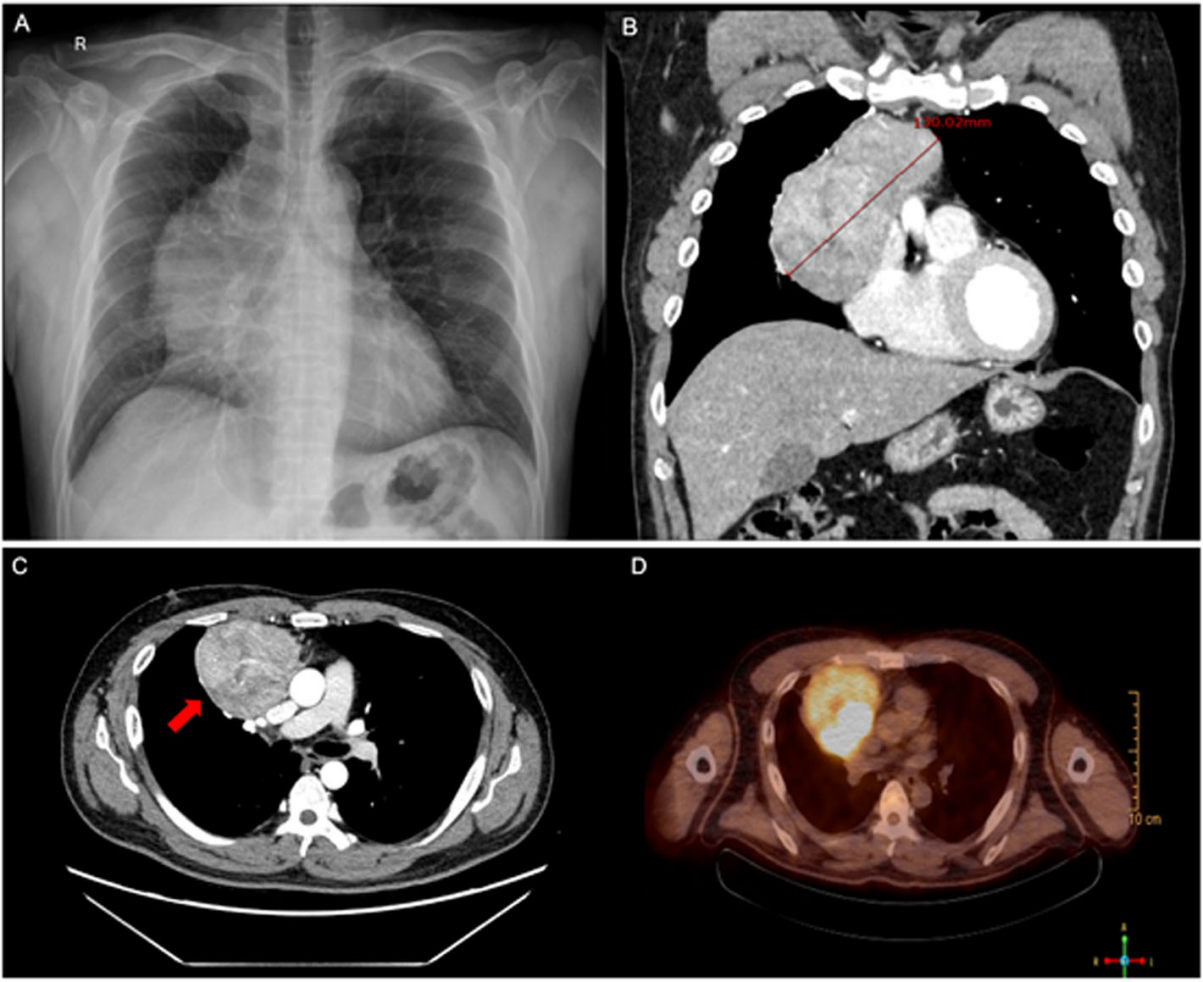
Images of the mediastinal mass. **a** Chest X-ray showing a lobulated right cardiomediastinal contour. **b** Contrast enhanced computed tomography (CT) showing a 13-cm-sized mediastinal mass in the sagittal plane (red arrow). **c** CT showing an anterior mediastinal mass invading the superior vena cava and pericardium (red arrow). **d** Fluorodeoxyglucose (FDG) positron emission tomography showing intense FDG uptake within the mass, with an SUVmax of 10.1

A 43-year-old man was admitted to the hospital due to an enlarged mediastinum incidentally observed on chest x-ray during a routine health check-up (Fig. 1a). The patient was a 20 pack-year current smoker and had recently diagnosed with hypertension. His father died suddenly in his 40s for unknown reasons, but there was no history of cancer, or chronic disease in his family or relatives. There was no persional or family history of kidney stones as well. At admission, the patient was asymptomatic, and physical examination and vital signs were unremarkable; Swelling of the face, neck, or upper body and dyspnea or distended neck veins were also not observed. No cutaneous findings were reported.

Enhanced chest computed tomography (CT) showed an anterior mediastinal mass invading the superior vena cava and pericardium that measured approximately 13.0 × 8.0 × 6.0 cm in size (Fig. 1b and c). Fluorodeoxyglucose-positron emission tomography indicated a hypermetabolic (SUVmax =10.6) mediastinal mass without additional uptake by other organs or regional lymph nodes (Fig. 1d).

**Fig. 2:**
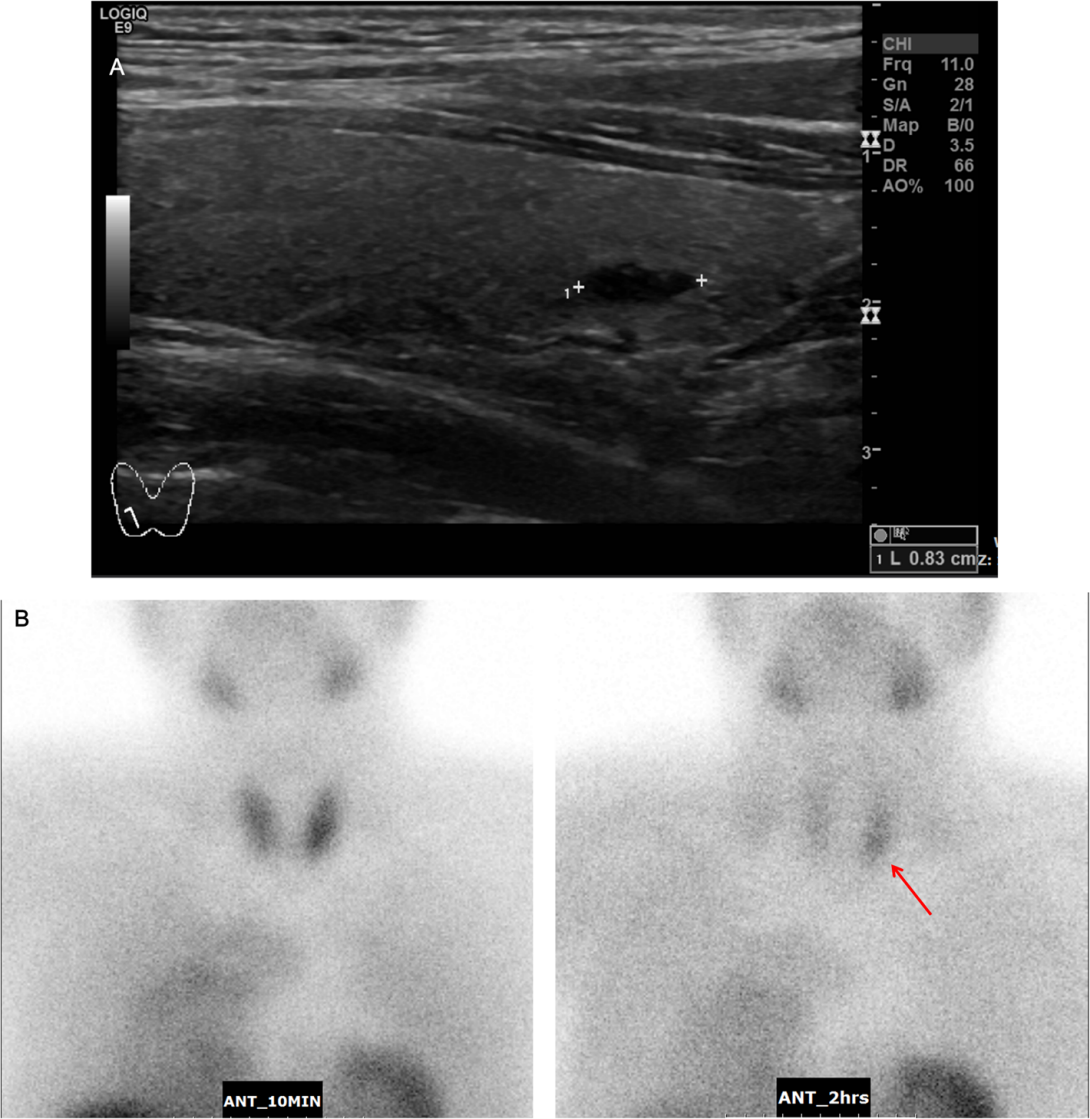
Images of the parathyroid glands**. a** The longitudinal scan of neck ultrasound demonstrates a well-defined hypoechoic solid mass posteroinferior to the left lobe of the thyroid gland. **b** Early and late 99 m Tc-sestamibi scintigraphy parathyroid scan images of the neck and anterior mediastinum at 10 min and 2 h, showing increased focal uptake (red arrow) suggestive of left inferior parathyroid adenoma

Laboratory results demonstrated elevated fasting plasma glucose of 128 mg/dL and HbA1c of 8.6%, which were sufficient to diagnose diabetes. Plasma insulin was 27.5 μU/mL (normal range, 1.4–12.4), fasting C-peptide was 4.85 ng/mL (normal range, 0.78–5.19), and glucagon was increased to 431.7 pg/mL (normal range, 25–250). Serum calcium level was slightly elevated at 10.9 mg/dL (normal range, 8.8–10.6) and albumin-corrected calcium was 10.8 mg/dL with a normal phosphorus level of 3.7 mg/dL (normal range, 2.5–4.5). In the work-up for hypercalcemia, serum intact parathyroid hormone (PTH) level was elevated at 78.7 pg/mL, and recheck level was more increased up to 128 pg/mL (normal range, 15–65). A 25-hydroxy vitamin D was low at 8.0 ng/mL, and the 24-h urine calcium amount was 300 mg/day. A bone density scan showed anterior-posterior spine L1–4 bone mineral density (BMD) of 0.806 g/cm^2^ with a Z-score of − 3.6, left femoral total BMD of 0.969 g/cm^2^ with a Z-score of 0.4, and left femoral neck BMD of 0.959 g/cm^2^ with a Z-score of 0.3. Neck ultrasound showed a 1.2 cm enlarged parathyroid gland (Fig. 2a) and a Tc-99 m sestamibi scan demonstrated focal increased activity at the left lower pole region (Fig. 2b), suggestive of functional parathyroid adenoma.

**Fig. 3:**
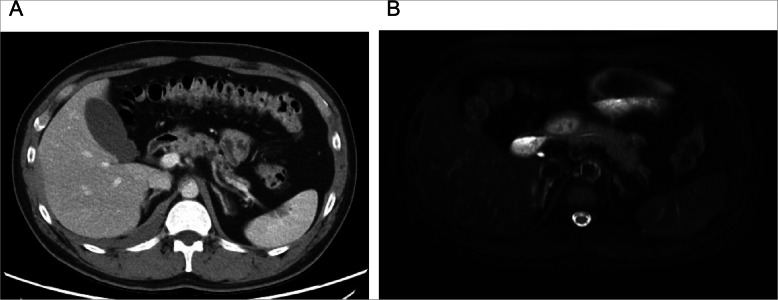
**a** Abdominal CT showing multiple small hypodense lesions in the head and body of the pancreas. **b** Magnetic resonance imaging demonstrates a normal pancreas

**Fig. 4:**
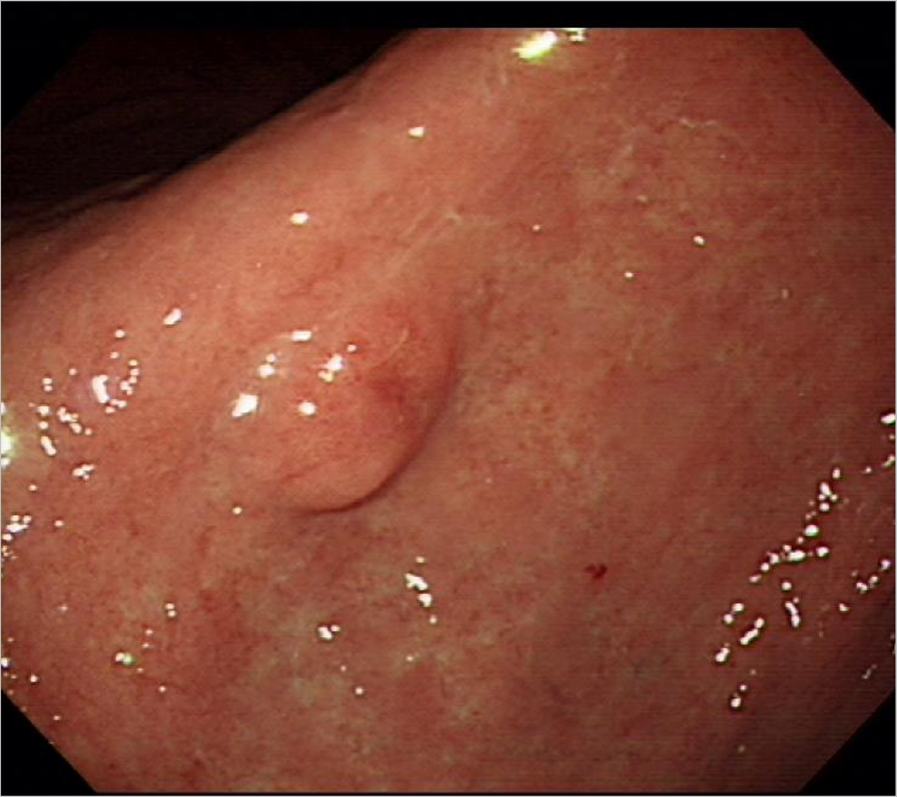
Endoscopic image of a gastric neuroendocrine tumor at the antrum revealed a well circumscribed, submucosal mass with normal overlying mucosa

Abdominal CT (Fig. 3a) found multiple small, low-attenuated lesions in the pancreatic head and body. However, there were no abnormal findings on magnetic resonance imaging (MRI) of the pancreas (Fig. 3b); thus, the pancreatic lesions observed on CT were considered peripancreatic fat infiltrations. Esophagogastroduodenoscopy (Fig. 4) demonstrated a submucosal mass at the antrum of the stomach, which was proven to be a well-differentiated neuroendocrine tumor (NET). Meanwhile, serum gastrin level was 54.9 pg/mL (normal range, < 180).

**Fig. 5:**
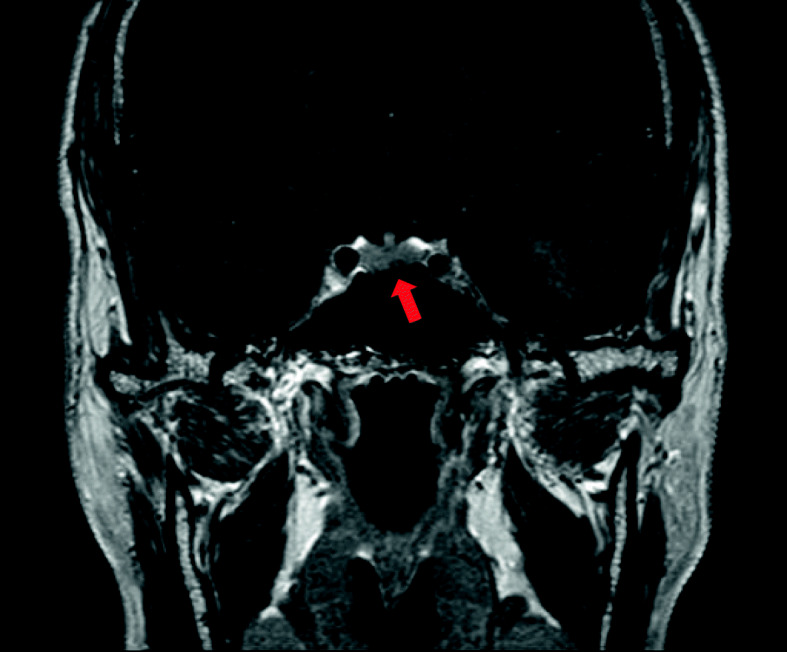
Sella MRI (coronal T1-weighted postcontrast) showing a 0.3-cm-sized, delayed enhancing lesion of the pituitary gland, suggestive of a pituitary microadenoma (red arrow)

Evaluations of the pituitary gland revealed a slightly elevated serum prolactin level of 21.9 ng/mL (normal range, 4.1–18.4), normal thyroid hormones, normal IGF-1, normal FSH, normal testosterone, but a low LH level < 0.5 mIU/mL (normal range, 0.57–12.07). Morning cortisol level was normal (10.2 μg/dL), and serum cortisol was suppressed by a 1 mg overnight dexamethasone. Basal ACTH level was 64.8 pg/mL (normal range, 4.7–48.8). Sella MRI showed a 0.3 cm, delayed enhancing lesion, which was hard to differentiate between pituitary microadenoma and normal variant of pituitary gland (Fig. 5).

Based on these results, the patient had thymic carcinoid tumor, primary hyperparathyroidism, and gastric NET as a result of MEN1. In genetic testing for MEN1 (Supplementary Fig. 1A and 1B), the patient and his 7-year-old son, but not his younger brother, had the same missense mutation (c.634G > A, p.Val215Met) of the *MEN1* gene.

**Fig. 6:**
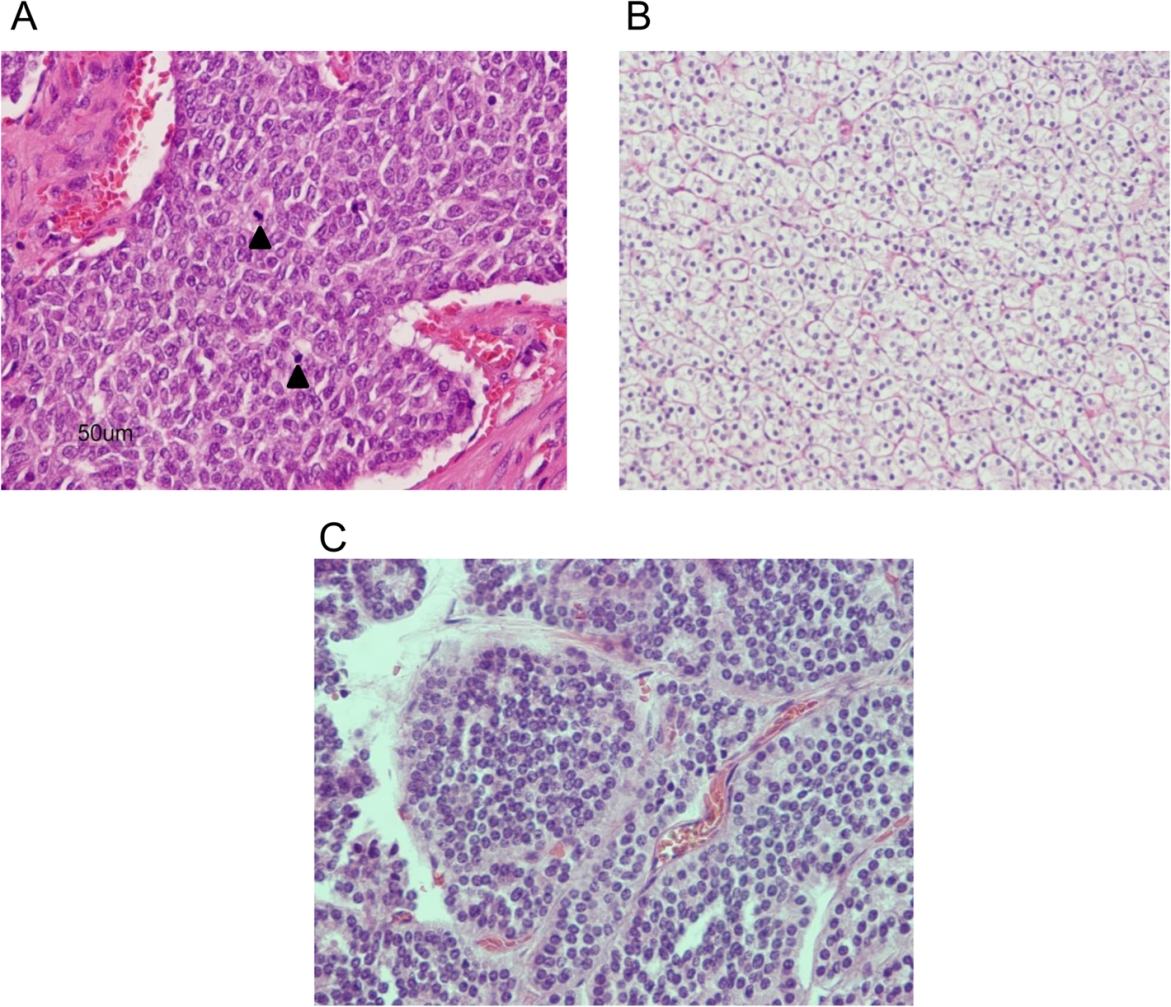
Pathological results of surgical resection. **a** Thymic cancer: The tumor exhibited a solid growth pattern with 9 mitoses per 10 high-power fields without necrosis (hematoxylin-eosin stain × 100). Black arrowhead indicates mitoses of carcinoid cells. **b** Water clear cells of parathyroid adenoma with minimal nuclear pleomorphism and have uniformly clear cytoplasm with numerous microvacuoles (hematoxylin-eosin stain × 100). **c** Gastric neuroendocrine tumor with salt-and-pepper chromatin, amphophilic cytoplasm, and scant atypia (hematoxylin-eosin stain × 100)

For treatment, neoadjuvant concurrent chemoradiotherapy reduced the size of the thymic carcinoid tumor. Finally, video-assisted thoracoscopic surgery on the thymic carcinoid and left inferior parathyroidectomy were simultaneously performed after 2 months of chemoradiotherapy. Postoperative pathology confirmed the final diagnosis of atypical thymic carcinoid (Fig. 6a) and parathyroid hyperplasia (Fig. 6b). The gastric NET was also removed by endoscopic resection (Fig. 6c). At one-year postoperative, there has been no tumor recurrence on follow-up chest CT, and serum calcium level and BMD returned to normal, though there was slight elevation in serum PTH level (72 pg/mL) after having been previously normalized (28.9 pg/mL). Serum prolactin level (20.3 ng/mL) did not change during the first postoperative year.

## Discussion and conclusions

This case was diagnosed with MEN1 on the basis of clinical, genetic and familial criteria (Supplementary Table 1) [2]. Interestingly, first manifestation of the patient was asymptomatic mediastinal mass by atypical thymic carcinoid tumor which is a rare entity in MEN1. MEN1 is an inherited tumor syndrome caused by inactivating heterozygous mutations of the *MEN1* gene that affect the parathyroid glands, neuroendocrine tissues of gastroenteropancreatic origin, thoracic tracts, and the anterior pituitary gland [6]. Parathyroid tumors, resulting in primary hyperparathyroidism, are the most common feature of MEN1 and occur in approximately 95% of patients, whereas pancreatic NETs occur in 40% and anterior pituitary tumors occur in 30% [6].

Thymic carcinoid tumor is generally a late manifestation of MEN1 [7]; in a previous report, it was most commonly diagnosed as an anterior mediastinal mass usually found on chest x-ray or CT scans during periodic health examination, consistent with this case [8]. In a different series of patients with MEN1-associated thymic carcinoid tumor, the age of presentation was between 30 and 50 years, the patients were predominantly heavy smokers, and the disease had a predilection for men over women [8, 9]. Despite its insidious onset, thymic carcinoid tumor is an important determinant of long-term survival in MEN1 patients, because MEN1-associated thymic carcinoid tumor leads to poor prognosis due to its more aggressive nature and strong potential for metastasis even after apparently radical excision [5, 8].

The unusual occurrence of MEN1 in this case was that the first presentation of the patient was asymptomatic huge thymic carcinoid, rather than a classical manifestation which is comprised of hyperplasia and/or tumors of parathyroid, pancreas, or anterior pituitary origin that occur at a high rate by the age 40 in the affected individuals [10]. Hypercalcemia was mild, although primary hyperparathyroidism is most common and early manifestation of MEN1. Moreover, the serum prolactin level of this patient was not high enough to suspect prolactinoma, and MRI imaging did not show the definite lesion. Pancratic lesions was also considered as benign fat infiltrations. Instead, he presented relatively rare MEN1-associated tumors that included thymic carcinoid (2% of estimated penetrance) and gastric NET (10% of estimated penetrance) [11], with *MEN1* mutation.

Treatment for each of the MEN1-associated endocrine tumors requires complete surgical resection, which is similar to treatment for respective tumors in non-MEN1 patients. However, the surgical outcomes of patients with MEN-1 associated tumors may not surpass those of patients with sporadic solitary tumors because MEN1-associated tumors simultaneously occur at multiple sites, have many occult metastatic lesions, and show more aggressive features [11]. Therefore, even prophylactic thymectomy should be considered in high-risk patients, such as male smokers or those with close relatives who present with this feature [8], to prevent development of thymic carcinoid tumor. Subtotal or total parathyroidectomy, definite treatment of primary hyperparathyroidism, is also recommended due to the high recurrence rate [11]. However, one overactive parathyroid gland in this patient was removed in the transcervical approach not to disturb the resection of huge mediastinal mass. The patient’s serum PTH and calcium levels have been annually monitored after parathyroidectomy in order to detect any possible recurrence from the remaining three parathyroid glands.

It is noteworthy that early diagnosis of MEN1 is mandatory for identification and subsequent treatment at a very early stage for preventing malignant progression and metastases as well as clinical consequences of hormone overproduction-related syndromes, which are the main causes of mortality and morbidity of patients with MEN1 [12]. In this respect, genetic and biochemical screening in asymptomatic family members of patients with MEN1 are likely to be beneficial for a longer survival [11]. *MEN1* mutation analysis should be undertaken in the index case, asymptomatic first-degree relatives of a known MEN1 carrier, and first-degree relatives of a *MEN1* mutation carrier expressing familial MEN1 [5]. The patient in the present case had a missense mutation of the *MEN1* gene (V215M on exon 3), which was reported in two prior MEN1 cases in Korea [13, 14]. DNA sequencing was performed for the patient’s 7-year-old son, and the same mutant gene was identified. Tumors have been reported in children with MEN1 by the age of 10 years [11], so we have been annually monitoring symptoms and performing biochemical tests on his son as well as the index patient.

In conclusion, the present study reported an asymptomatic MEN1-associated thymic carcinoid as the first manifestation of MEN1. All MEN1-associated endocrine tumors were successfully removed, and there was no evidence of tumor recurrence at postoperative 1 year. Systematic evaluations and close follow-up of other endocrine organs are needed in patients with thymic carcinoid tumor and *MEN1* mutation.

## Supplementary Information

**Additional file 1: Supplementary Fig. 1.** (A) Pedigree chart of the patient. The index patient and his son carry the same *MEN1* gene mutation. (B) DNA sequencing shows a heterozygous missense mutation in exon 3 (p.Val215Met).

**Additional file 2: Table S1**. Diagnostic criteria of MEN1 *[2]

## Data Availability

The datasets used and/or analyzed during the current study are available from the corresponding author on reasonable request.

## Abbreviations

*MEN1*: Multiple endocrine neoplasia type 1
*CT*: Computed tomography
*PTH*: Parathyroid hormone
*BMD*: Bone mineral density
*MRI*: Magnetic resonance imaging
*NET*: Neuroendocrine tumor
