# Supplementary figures and images for: A rare case of multiple endocrine neoplasia type 1 initially presenting as an asymptomatic, huge mediastinal mass: case report
Source: BMC Endocr Disord. 2021 Feb 25;21:31. doi: 10.1186/s12902-021-00695-9 (PMC7905909; doi:10.1186/s12902-021-00695-9)

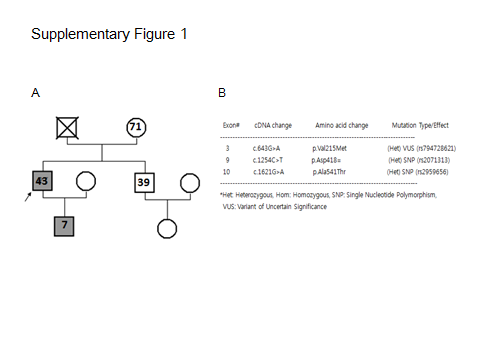

Supplement: Supplementary file 1 — Additional file 1: Supplementary Fig. 1. (A) Pedigree chart of the patient. The index patient and his son carry the same MEN1 gene mutation. (B) DNA sequencing shows a heterozygous missense mutation in exon 3 (p.Val215Met). [file 12902_2021_695_MOESM1_ESM.docx]
