# Supplementary material for: A rare case of multiple endocrine neoplasia type 1 initially presenting as an asymptomatic, huge mediastinal mass: case report
Source: BMC Endocr Disord. 2021 Feb 25;21:31. doi: 10.1186/s12902-021-00695-9 (PMC7905909; doi:10.1186/s12902-021-00695-9)
Supplement: Supplementary file 2 — Additional file 2: Table S1. Diagnostic criteria of MEN1 *[2] [file 12902_2021_695_MOESM2_ESM.docx]

Table S1. Diagnostic criteria of MEN1*[2]

| 1. Two or more MEN1-associated endocrine tumors as below 2. Parathyroid adenoma 3. Enteropancreatic tumor: gastrinoma, insulinoma, glucagonoma, VIPoma, Somatostatinoma, non-functioning and PPoma. 4. Anterior pituitary adenoma : prolactin-, GH-, ACTH- secreting adenoma, nonfunctioning adenoma 5. Other tumors : foregut carcinoid tumors (thymic, bronchial, gastric NET), adrenocortical tumors, cutaneous tumors, meningiomas, ependymomas, tumors of smooth muscle and very rarely pheochromocytoma. |
| --- |
| 1. One of the MEN1-associated tumors in a first-degree relative of a patient with a clinical diagnosis of MEN |
| 1. Germline *MEN1 mutation* in an individual who may be asymptomatic and has not yet developed serum biochemical or radiological abnormalities indicative of tumor development. |

*MEN1 patient must fulfill at least one of three criteria.

MEN1: multiple endocrine neoplasia type 1, VIP: vasoactive intestinal polypeptide PP: pancreatic polypeptide, GH: growth hormone, ACTH: Adrenocortical tropic hormone. NET: neuroendocrine tumor.
